# Bacterial aerobic methane cycling by the marine sponge-associated microbiome

**DOI:** 10.1186/s40168-023-01467-4

**Published:** 2023-03-10

**Authors:** Gustavo A. Ramírez, Rinat Bar-Shalom, Andrea Furlan, Roberto Romeo, Michelle Gavagnin, Gianluca Calabrese, Arkadiy I. Garber, Laura Steindler

**Affiliations:** 1 Department of Marine Biology, Leon H. Charney School of Marine Sciences, University of Haifa, 199 Aba Khoushy Ave., Mount Carmel, Haifa, Israel; 2 Present address: Department of Biological Sciences, California State University, Los Angeles, CA USA; 3 Istituto Nazionale di Oceanografia e di Geofisica Sperimentale, Trieste, Italy; 4 School of Life Science, Arizona State University, Tempe, AZ USA

**Keywords:** Methane, Porifera, Microbiome, Aerobic methane synthesis, Methylphosphonate, Methylamine, Candidate phylum Binatota, *Aplysina aerophoba*, *Petrosia ficiformis*

## Abstract

**Background:**

Methanotrophy by the sponge-hosted microbiome has been mainly reported in the ecological context of deep-sea hydrocarbon seep niches where methane is either produced geothermically or via anaerobic methanogenic archaea inhabiting the sulfate-depleted sediments. However, methane-oxidizing bacteria from the candidate phylum Binatota have recently been described and shown to be present in oxic shallow-water marine sponges, where sources of methane remain undescribed.

**Results:**

Here, using an integrative *-omics* approach, we provide evidence for sponge-hosted bacterial methane synthesis occurring in fully oxygenated shallow-water habitats. Specifically, we suggest methane generation occurs via at least two independent pathways involving methylamine and methylphosphonate transformations that, concomitantly to aerobic methane production, generate bioavailable nitrogen and phosphate, respectively. Methylphosphonate may be sourced from seawater continuously filtered by the sponge host. Methylamines may also be externally sourced or, alternatively, generated by a multi-step metabolic process where carnitine, derived from sponge cell debris, is transformed to methylamine by different sponge-hosted microbial lineages. Finally, methanotrophs specialized in pigment production, affiliated to the phylum Binatota, may provide a photoprotective function, closing a previously undescribed C_1_-metabolic loop that involves both the sponge host and specific members of the associated microbial community.

**Conclusion:**

Given the global distribution of this ancient animal lineage and their remarkable water filtration activity, sponge-hosted methane cycling may affect methane supersaturation in oxic coastal environments. Depending on the net balance between methane production and consumption, sponges may serve as marine sources or sinks of this potent greenhouse gas.

Video Abstract

**Supplementary Information:**

The online version contains supplementary material available at 10.1186/s40168-023-01467-4.

## Background

Sponges, globally dispersed sessile metazoans, host vastly diverse microbiomes [1] in a complex association collectively recognized as the sponge “holobiont” [2]. The pre-Cambrian fossil record [3, 4] and recent phylogenetic analyses [5] suggest that the sponge holobiont represents the most ancient of extant metazoan-microbe interactions [6]. The high-volume filter feeding of marine sponges [7] links benthic and pelagic biogeochemical processes and influences major nutrient cycles in diverse ocean ecosystems [8–13]. High microbial abundance (HMA) sponges may derive up to 35% of their mass from microbial symbionts [14, 15], accounting for a 1000-fold higher microbial load than the surrounding seawater [16]. The sponge microbiome is also responsible for producing, largely unclassified, secondary metabolites of biotechnological interest [17].

Intriguingly, sponge species seem to share a small core microbiome while hosting a large species-specific community [1]. Eukaryote-like repeat proteins, CRISPR-Cas systems, and DNA phosphorothioation are important mediators of symbiont-host recognition and defense against non-symbionts and viruses [18–22]. Some Thaumarchaeota are keystone symbionts that oxidize host-excreted ammonia [23–25], thereby preventing the toxic effects of this exudate on the sponge host [26]. Physiological studies have addressed the cycling of carbon [8, 27] and nutrients (nitrogen [24, 28, 29], phosphorus [30, 31], silicon [32]) by the sponge holobiont using both indirect and direct [33, 34] techniques, resulting in a better understanding of the holobiont functioning, its nutrient budgets, and the related ecological impact (reviewed in [35, 36]). Stable isotope incubations combined with imaging techniques (Nano-SIMS) have revealed that not only microbial symbionts but also host choanocyte sponge cells can directly take up dissolved organic matter (DOM) and that it is the latter host cells that first assimilate DOM from filtered seawater, likely via pinocytosis, and then translocate the processed DOM to the symbionts inhabiting the sponge mesohyl [37, 38].

Recent efforts have characterized the community-level functional potential of sponge symbionts [18, 39] and significantly increased the number of available sponge-associated microbial genomes [40]. Genome-centered metatranscriptomic sponge surveys have explored host-microbe interactions, energy and carbon metabolism [41, 42], nitrogen cycling [24, 43], and, recently, the Tethybacterales: an uncultured clade within the Gammaproteobacteria [44]. Despite these advances, few studies have linked sponge biogeochemical cycling activities to the corresponding microbial lineages or described how these activities may influence, over geological time scales, the biogeochemical state of the planet [45]. For example, the extent to which sponges are involved in the cycling of short-chain alkanes is underexplored.

The co-detection of archaeal methanogens and sulfate-reducing bacteria (SRBs) long ago suggested the presence of anaerobic niches in demosponge tissue [46]. Later work showed that sponge pumping dynamics [47] as well as distinct oxygen removal patterns not related to water pumping activity [48] elicit anoxic microenvironments where active anaerobic microbial activities such as sulfate reduction [49], denitrification, and fermentation [50] occur. Hydrocarbon degradation by sponge symbionts in deep-sea seep environments has previously been characterized and involves methylotrophic and short-chain alkane (methane, ethane, butane) specialized symbioses [51–54]. This body of work indicates that canonical archaeal methanogenesis, in anoxic tissue microniches, and short-chain alkane oxidation, in hydrocarbon-rich deep-sea hydrothermal vent and cold seep environments, are activities present in sponge-associated microbiomes. Interestingly, methane oxidation activity is also predicted for sponge-associated members of the proposed candidate phylum Binatota [55] (also annotated as Desulfobacterota) hosted by *Petrosia ficiformis*, a sponge inhabiting fully oxygenated shallow seas where no known hydrothermal venting or hydrocarbon seepage exists [42]. The presence of Binatota (described as Deltaproteobacteria bin18) was also reported in the shallow-water growing sponge species *Aplysina aerophoba* [39]. The source of methane for methanotrophs hosted by these oxic shallow-water marine sponges remains unknown.

Here, activities of the *A. aerophoba*-hosted microbiome, residing in fully oxic seas where no hydrocarbon seepage or hydrothermal venting is known, were explored using genome-centered metatranscriptomics, gene-targeted sequencing, and metabolomics. We find no evidence for canonical archaeal methanogenesis and show that aerobic bacterial methane synthesis may occur via two recently described metabolisms: (i) methylphosphonate (MPN) degradation, the proposed solution to the ocean’s methane paradox [56, 57], and (ii) a recently described methylamine (MeA) [58] transformation catalyzed by a 5′ pyridoxal-phosphate-dependent aspartate aminotransferase [59]. Furthermore, we report that microbial community processing of cell debris generates carnitine. Carnitine may be subsequently metabolized to MeA and ultimately methane. We highlight that a potential fate of this biogenic methane, produced endogenously through either the MeA or MPN pathways, is oxidation by methylotrophic members of the candidate phylum Binatota, a lineage specialized in the production of photoprotective pigments that may benefit the host and, thereby, describe a novel methane-centered “metabolite processing loop” of potential symbiotic importance.

## Methods

### Sampling and sample preservation

Mediterranean *Aplysina aerophoba* specimens were sampled from the Northern Adriatic in the Gulf of Trieste (45°36.376, 13°43.1874), using SCUBA, within meters of each other. Four individual sponge specimens were separately sampled twice in a 24-h period (at 12:00 noon day 1 and 12:00 noon day 2). Immediately upon collection, all tissues were in situ preserved in RNA*Later*® (Sigma-Aldrich) solution using an underwater chamber as detailed elsewhere [60] and, once out of the water, kept on ice for a few hours prior to freezing and transport to shore-based storage at −80°C.

### Preparation of libraries and metatranscriptomic sequencing

RNA was extracted using Allprep DNA/RNA mini kit (Qiagen, Germany). Briefly, each extraction was performed using 30 mg of sponge sample placed in a Lysing Matrix E tube (MP Biomedicals, Santa Ana, CA) to which RLT buffer containing Reagent DX (Qiagen, Hilden, Germany) was added. Cells were disrupted using a TissueLyser II system (Qiagen, Germany) for 30 s at 30 Hz followed by 10-min centrifugation at maximum speed. All subsequent RNA extraction steps were performed according to the manufacturer’s protocol. SUPERase In (Life Technologies, USA) and TURBO DNA-free kit (Thermo Fisher Scientific, USA) were used for RNase inhibition and DNase treatments, respectively. RNA cleanup and concentration were done using the RNeasy MinElute kit (Qiagen, Germany). In order to achieve sufficient coverage of informative nonribosomal transcripts, rRNA was removed with a RiboMinus Eukaryote System V2 kit (Ambion, Life Technologies, USA) with eukaryotic mouse-rat-human probes coupled with prokaryotic probes. ERCC RNA Spike-In Control mixes (Life Technologies, USA) were added to 5 µg of total RNA. RNA concentrations were measured using a Qubit 2.0 Fluorometer and RNA reagents (Thermo Fisher Scientific, USA), before and after rRNA depletion. In parallel, RNA integrity and purity were determined using a TapeStation 2200 system, applying the High sensitivity RNA Screen Tape assay (Agilent Technologies, USA), before and after rRNA depletion as well. Ultimately, 13ng of rRNA-depleted RNA was processed for cDNA library preparations using the Collibri stranded RNA library prep kit (Thermo Fisher Scientific, USA) according to the manufacturer’s protocol with the sole exception being that, following the addition of the index codes, cDNA amplification was performed with 8 rather than the 9–11 recommended PCR cycles. The number of PCR cycles was optimized for our samples to reduce PCR bias. The libraries were quantified using Invitrogen Collibri Library Quantification Kit (Invitrogen, Thermo Fisher Scientific) according to the manufacturer’s guide using real-time qPCR. For pre-sequencing quality control (QC), 2-μl aliquots of each provided library were pooled. The resulting QC pool was size and concentration checked on an Agilent D1000 TapeStation system and a Qubit 2.0 fluorometer, respectively. The pool was adjusted to 1nM and loaded on an Illumina MiniSeq Mid Output flow cell at 1.5pM. After demultiplexing, the percent of each library was used to calculate new volumes to use for constructing a normalized sequencing pool. This pool was also size and concentration checked, as described above, and subsequently normalized to 2nM. The normalized pool was run on an Illumina NextSeq High Output flowcell at 2.2pM.

### Metatranscriptomic sequence processing

Paired-end Illumina libraries were inspected for quality parameters and repetitive sequences using the FastQC software package. Adapter trimming was performed using the trim adapters bbduk script from BBMaps (https://sourceforge.net/projects/bbmap/). Trimmed paired-end files were interleaved for alignment against rRNA libraries using SortMeRNA [61]. Non-aligned reads were subsequently split into paired forward and reverse files for downstream analyses. A de novo co-assembly was performed using merged forward and reversed adapter trimmed and non-rRNA aligned sequences with rnaSPAdes v.3.14.1 [62]. Sequence counts at each step for all libraries, in addition to co-assembly summary statistics, are provided in Table S1.

### 16S rRNA gene and transcript ASV analysis

As detailed above, RNA and DNA were extracted in parallel and RNA was subsequently reverse transcribed. DNA and cDNA extracts were used as templates for PCR-based amplification for 16S rRNA gene/transcript V4 amplicon generation using the following primers: 515F-ACACTGACGACATGGTTCTACAGTGCCAGCMGCCGCGGTAA and 806R-TACGGTAGCAGAGACTTGGTCTGGACTACNVGGGTWTCTAAT, and thermocycling program: 94°C for 3 min, followed for 32 × [ 94°C for 45s, 50°C for 60s, 72°C for 90s], 72°C for 10 min, and a 4°C hold. Amplicon sequencing was performed on an Illumina MiSeq platform. Sequence analyses were performed using the DADA2 package [63] implemented in R. Briefly, forward and reverse reads were trimmed with the filterAndTrim() command using the following parameters: trimLeft = c(20,20), maxEE = c(2,2), phix = TRUE, multithread = TRUE, minLen = 120, followed by error assessments and independent forward and reverse read de-replication. Sequencing errors were removed using the dada() command and error-free forward and reverse reads were merged using the mergePairs() command, specifying overhand trimming and a minimum overlap of 120 base pairs. The resulting amplicon sequence variants (ASVs) were assigned taxonomy by alignment against the SILVA 132 database [64]. ASV count tables and taxonomy assignments were merged into an S4 object for diversity analysis and summary visualization using vegan in phyloseq [65].

### Pathway completion estimates

Prodigal v2.6.1 [66] predicted protein products were annotated against the KEGG database [67] via GhostKOALA [68] with the following parameters: taxonomy group, Prokaryotes; database, genus_prokaryotes + family_eukaryotes; accessed February 2021. The output annotation file was used for pathway completion assessment and visualization using KEGG-decoder [69].

### Read mapping

To assess coverage as a proxy for transcript abundance, quality-trimmed non-rRNA short reads were mapped to our de novo metatranscriptomic assembly and a public reference set of metagenome assembled genomes (MAGs) binned from *A. aerophoba* in close geographical proximity to our study site [39] using Bowtie2 [58]. Read counts were normalized to Transcripts per Million (TPM) per library, as suggested elsewhere [70], and all data was concatenated into read count tables for downstream statistical analyses.

### Phylogenomic tree

A phylogenomic tree was generated for *A. aerophoba-*derived MAGs using the GToTree package [71]. Briefly, 37 publicly available MAGs [39] were used as the input and were ran against a GToTree’s “Bacteria” HMM collection of single copy genes within this domain resulting in a concatenated protein alignment constructed using Muscle [72] and trimmed with TrimAl [73]. The tree was constructed in FastTree2 [74] and visualized using FigTree (https://github.com/rambaut/figtree).

### ORF calling, annotation, and targeted gene analyses

Open reading frame (ORF) identification and, subsequently, prokaryote-predicted protein product annotations were performed with Prodigal v2.6.1 [66] implemented in Prokka [75]. Selected ORFs were also aligned against the NCBI non-redundant (nr) database (accessed in April 2021) using BLASTp for closest homologue taxonomy and functional annotation supplementation. Targeted single gene homologue searches within our data were also performed using BLASTp 2.2.30+ (*E*-value threshold = 1E−30, identity = 50%) against predicted protein sequences inferred from (i) metatranscriptomic assemblies, (ii) unbinned metagenomic contigs, and (iii) MAGs.

### Metabolome analysis

Four ~1.5-g frozen tissue samples collected from specimens 25, 27, 28, and 29 each in 1:1 wt:wt sample to EtOH ratio solutions were shipped on dry ice for biogenic amines panel metabolome analyses by HILIC-QTOF MS/MS [76] at the UC Davis West Coast Metabolomic Center (http://metabolomics.ucdavis.edu/). Raw output files were curated based on internal standard removal, signal to noise ratio cutoff, and a minimum peak threshold of 1000 and subsequently parsed based on average (*n*=4) metabolite log fold increases relative to blanks, as suggested elsewhere [77]. Only metabolites with non-redundant names and InChIKey identifiers and average fold changes higher than 5 relative to blanks were retained for further analyses.

## Results and discussion

### Net relative activity of symbiont lineages

**Fig. 1:**
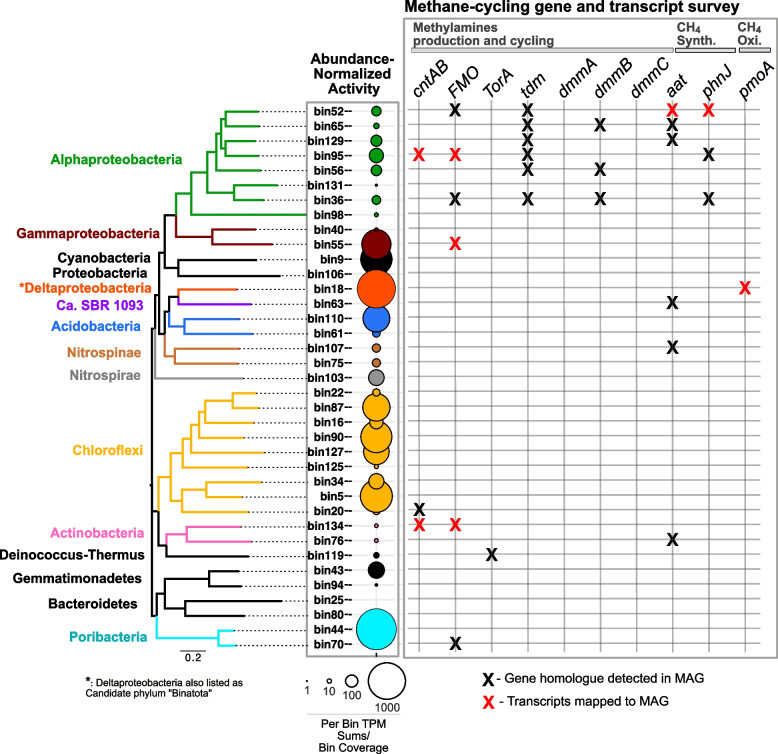
Survey of methane cycling genes and transcripts. Left—relative global activity survey for 37 dominant lineages, arranged based on phylogenomic relatedness and color-coded at the phylum-level of taxonomy, in the *A. aerophoba*-associated microbiome. Relative activity is represented by circle size and depicts mean values per lineage from eight independent metatranscriptomes: four sponge specimens sampled twice, at noon 24 h apart (see Fig. S2 for individual results). Right—methane-relevant genes and gene transcripts detected in MAGs and in the metatranscriptomes are depicted by black and red “X” symbols, respectively

A normalized activity survey based on metatranscriptomic read mapping against 37 MAGs [39] representative of the *A. aerophoba*-associated microbial community (Fig. S1) shows Gammaproteobacteria, Cyanobacteria, Deltaproteobacteria, Acidobacteria, Chloroflexi, and Poribacteria as the most active lineages (Fig. 1, Fig. S2). The Alphaproteobacteria appear to be an abundant and diverse lineage with relatively lower levels of activity. Our observations corroborate previous work regarding sponge microbiome activity [43] and highlight consistent transcriptional activity, i.e., the relative activity of each symbiont lineage is constant across time (24h) and is similar in magnitude in different sponge specimens (*n* =4, Fig. S2).

### Sponge microbiome predicted activities: C, N, and S cycling, fermentations, and photosystems

**Fig. 2:**
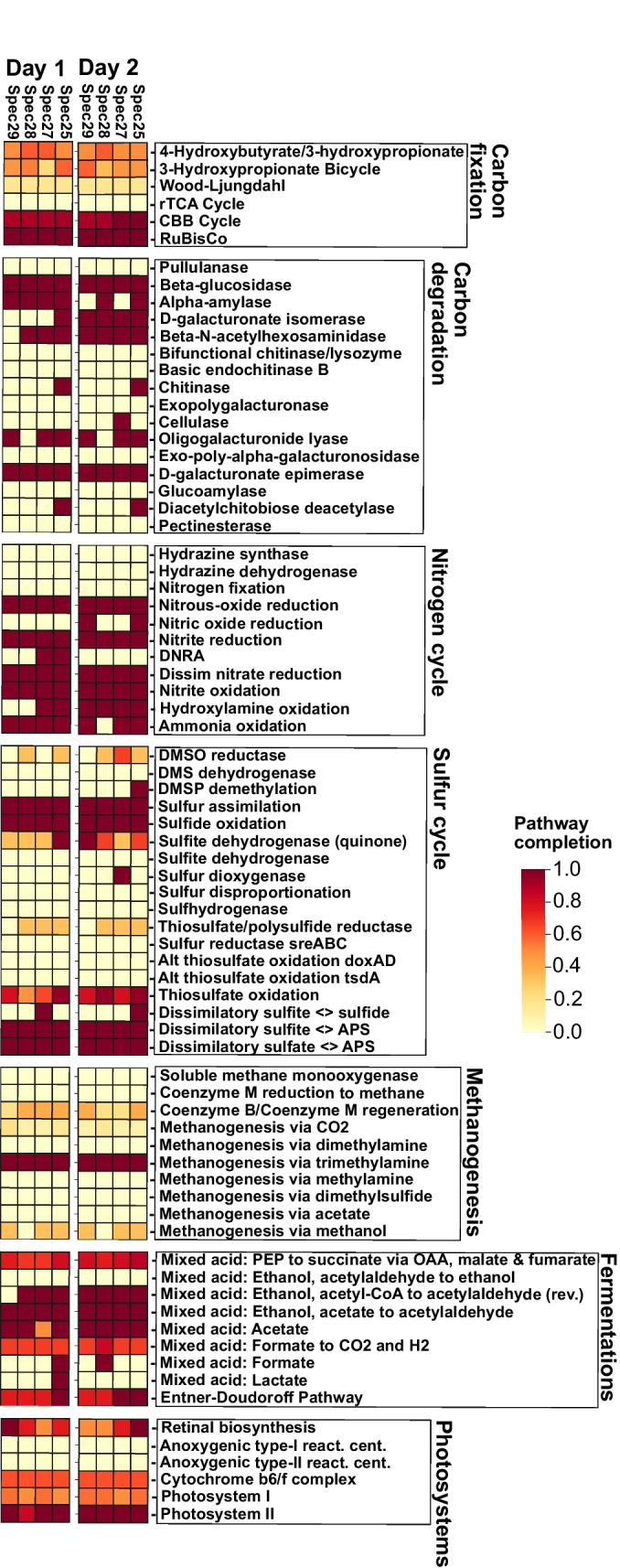
Microbiome metatranscriptome-predicted activity. Microbial activity survey based on collection day and specimen source for selected microbial metabolisms: carbon fixation, carbon degradation, nitrogen cycle, sulfur cycle, methanogenesis, fermentation, and photosystems. Day 1 and day 2 depictions represent biological replicates collected 24h apart at noon. The color gradient depicts the fractional percentage of KEGG module pathway completion

As a broad metatranscriptomic-predicted activity survey of the *A. aerophoba*-associated microbiota, aerobic and anaerobic metabolic pathways [49] were explored (Figs. 2 and S3). Carbon fixation via oxygenic photoautotrophy (CBB cycle) predominates. The incomplete transcription of other potential chemoautotrophic pathways [e.g., 3-hydroxypropionate (3HP) bicycle, and 4-hydroxybutyrate/3-hydroxypropionate (4HB/3HP)] is also observed. Previous transcriptomic and metatranscriptomic surveys report the presence of CBB and rTCA cycles in Demosponge-associated symbionts [41, 78]. Here, we corroborate an active CBB cycle, but fail to detect transcripts associated with the rTCA pathway suggesting that the rTCA cycle may not be a ubiquitous feature across sponge microbiomes.

Active carbon degradation pathways include those of pectin, chitin, cellulose, D-galacturonate epimerase, D-galacturonate isomerase, oligogalacturonide lyase, chitinase, cellulase, and alpha-amylase utilization. The catalysis of plant cell walls (pectin and cellulose) and continuously regenerated sponge matrix components (chitin) posits heterotrophy as a functional motif for this symbiosis, as previously suggested [79]. Interestingly, diacetylchitobiose deacetylase activity is a chitin degradation pathway only found in Archaea [80] and shows the heterotrophic activities of these often overlooked community members (Fig. S3).

An active sulfur cycle is predicted in our samples (Fig. 2), adding to a growing body of evidence showing that microbial sulfur cycling is an important sponge resource [81–83]. Continuous sulfur cycling, localized to anoxic niches within sponges, may benefit the host by the removal of toxic metabolites such as hydrogen sulfide [49, 84]. Diversification of sulfur metabolism in bacteria coincides with the emergence of metazoan life [85], suggesting a long co-evolutionary history reflected in the reduced genomes, as reviewed elsewhere [86], and versatile metabolisms of contemporary sulfur cycling sponge symbionts [82, 83].

Transcripts related to acetate and ethanol-based mixed acid fermentations are detected and suggest activities with reactions obligatorily localized to anaerobic niches [49]. Complete pathways for thiamin, riboflavin, and cobalamin synthesis (vitamins B_1_, B_2_, and B_12_, respectively) and for all 20 essential amino acids and other B vitamins, important microbial products with potential host benefits, are expressed across all biological replicates (Fig. S3) and support metabolite exchange [87] as one potential driver for this symbiosis.

Photoactivity is dominated by cyanobacterial Photosystem II transcripts and corroborates previous reports [60, 88]. Photosynthate transport from symbiotic photoautotrophs to the sponge host has been suggested as a common activity underpinning this symbiosis [60, 88, 89] but the transfer of photosynthates to the sponge is host- and cyanobacteria-specific and cannot be predicted by -*omics* data alone [42].

Interestingly, we detected a transcribed predicted pathway for methanogenesis via trimethylamine (TMA) (Fig. 2), a known activity of archaea in the human gastrointestinal tract [90]. Nitrogen cycling activity was also expressed and is described and discussed later in relation to the observed prediction of methane production pathways.

### Canonical anaerobic archaeal methanogenesis is absent in *A. aerophoba*

Motivated by the presence of methanotrophs (Binatota) and by the unexpected KEGG-pathway prediction for TMA-based methanogenesis (Fig. 2), we used a combined -*omics* approach to explore methane production avenues in *A. aerophoba*. We do not detect archaeal methanogenic lineages canonically associated with this activity (Fig. S1) nor genes or transcripts for euryarchaeal methyl-coenzyme M reductase (*mcrA*). Additional scrutiny of transcripts encoding TMA methyltransferase homologues (*mttB* within the COG5598 super family) revealed that these genes lack pyrrolysine (Fig. S4), a characteristic non-canonical amino acid residue involved in methane cleavage from methylated amines [91]. Non-pyrrolysine *mttB* homologues allow the strict anaerobe *Desulfitobacterium hafniense* to grow on glycine betaine with carbon dioxide and dimethylglycine as by-products [92]. Accordingly, we hypothesize that these *mttB* homologues may still play an important role in TMA cycling; however, the functional prediction of TMA methylotrophic methanogenesis by Euryarchaeal *mttB* is likely incorrect. Accordingly, alternative methane sources for Binatota methanotrophy in *A. aerophoba* were investigated.

### Sponge-hosted aerobic bacterial methane synthesis: marine methane paradox

Studies of the oversaturation of methane in fully oxygenated aquatic environments, a phenomenon dubbed the “marine methane paradox” [93] in the ocean, have revealed that aerobic bacterial degradation of methylphosphonates [56] and methylamines [59] results in methane production. These observations challenge the view of methanogenesis as a strictly anaerobic process performed exclusively by archaea and show that (i) bacteria play an important role in the production of a potent greenhouse gas and that (ii) this activity occurs in a broader range of redox (e.g., aerobic, microaerophilic) and chemical (sulfidic) environments. We, thus, searched for the presence and expression of marker genes for these aerobic methane synthesis pathways in sponge-derived MAGs [39] and our metatranscriptomes.

### Methylphosphonate-based aerobic bacterial methane synthesis

An alternative pathway for methane generation is the aerobic degradation of methylphosphonates (MPNs) through the C-P lyase activity of PhnJ [56]. This pathway is enriched in free-living marine pelagic bacteria from phosphate-limited locations including the Sargasso and Mediterranean Seas [94], the latter being the sampling site of our sponge specimens. Genes encoding *phnJ* homologues are transcribed across all our metatranscriptomic libraries with mean transcription values across specimens of 43.078±18.523 and 35.683±14.974 *recA* normalized TMP*1000 for the Noon1 and Noon2 sampling points, respectively (data not shown). We also detect *phnJ* gene homologues in 3 Alphaproteobacterial MAGs, with active transcription of this gene detected in at least one lineage (bin52, Fig. 1). Phosphonate metabolism was previously reported for the sponge *Xestospongia muta* [81] which, together with our results, suggests that seawater-derived phosphonates may be an important source of inorganic phosphate for sponge-associated microbes. An additional mechanism for phosphate sequestration in phosphate-limited conditions is the production of polyphosphates, which was previously reported for sponge symbionts [13]. Overall, PhnJ activity suggests that sponge symbionts may experience phosphate-limited conditions and that mechanisms to cope with this nutrient limitation are important for symbiont survival. We highlight that these results also imply that hydrocarbons, including methane, are potentially released as a result of phosphonate cleavage by C-P lyase.

### Methylamine-based aerobic bacterial methane synthesis

**Fig. 3:**
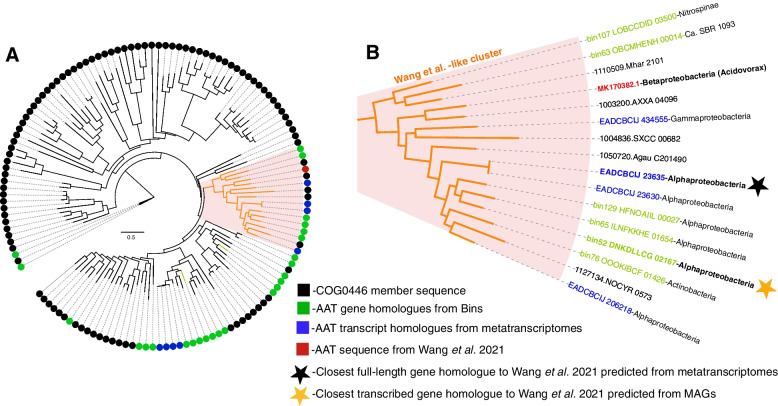
Phylogeny of *aat* genes and transcripts. **A** Phylogenetic tree of 100 COG0446 member sequences (black leaves) including *aat* gene (green leaves) and transcript (blue leaves) homologues identified from *A. aerophoba* MAGs and metatranscriptomes, respectively. The closest sponge-recovered gene and transcript sequences to MK170382.1 (red leaf), the *aat* gene confirmed to confer the methanogenesis phenotype in *E. coli* by Wang and colleagues [59], are emphasized in the orange cluster. **B** A focused depiction of the Wang and colleagues cluster highlighting the phylogenetic provenance of all highly similar sequences to MK170382.1 and showing the closest full-length genes recovered from MAGs and metatranscriptomic assemblies with gold and black stars, respectively

Recently, a new MeA-based aerobic methane synthesis metabolism, using 5′ pyridoxal-phosphate-dependent aspartate aminotransferase, was described in freshwater bacteria [59]. We report the transcription in our metatranscriptomic libraries of closely related gene homologues to the functionally confirmed proteobacterial *aat* (sequence: MK170382.1 recovered from *Acidovorax* sp.), the single heritable unit required to confer methane generating activity to an *E. coli* clone (Fig. 3). The closest transcribed homologues to MK170382.1, classified as Alphaproteobacterial predicted proteins, also maintain conserved key functional domains: a catalytic Lys residue and nine pyridoxal 5′-phosphate binding sites (Figs. 3 and S5). See supplemental materials for a version of Fig. 3A with labeled leaves (Fig. S6). Additionally, *aat* gene homologues detected in MAGs allowed the assignment of Alphaproteobacteria, Actinobacteria, Nitrospinae, and SBR phylogenetic provenance. At least one of these lineages (Alphaproteobacterial bin52) actively transcribes this gene (Figs. 1 and 3). Noting that AAT expressed in *E. coli* confers aerobic methane synthesis ability in the presence of MeA [59], our genomic and metatranscriptomic findings suggest that demosponges may host aerobic bacterial methane synthesis via AAT-mediated MeA metabolism. Methane generation via *aat* expression is also linked to growth in *Acidovorax* and when heterologously expressed in *E. coli*, suggesting that methanogenic activity may concomitantly allow anabolic nitrogen uptake. *aat-*based methane release from MeA rather than the oxidation of the MeA-methyl groups for energy may only be favored under high carbon and low nitrogen conditions. While sponge-secreted ammonia may result in bioavailable nitrogen to the associated microbiota, conditions of nitrogen availability inside the sponge are variable. For example, the fluxes of dissolved inorganic nitrogen differed across specimens of *X. muta*, with some specimens serving as sources and others as sinks of dissolved inorganic nitrogen [87, 95]. Furthermore, *A. aerophoba* was shown to serve as an ammonium sink in spring and as an ammonium source in the fall [24]. Some sponge species are characterized by constitutive nitrogen fixation [96] which may provide continuous bioavailable nitrogen to the microbial community; however, in the case of *A. aerophoba*, we could not detect genes involved in nitrogen fixation based on our metatranscriptomics predictions (Fig. 2). Finally, while our metatranscriptomic analysis predicts ammonia oxidation coupled with nitrite oxidation, as well as dissimilatory nitrate reduction (i.e., nitrate ammonification), it also predicts denitrification, which results in loss of bioavailable nitrogen sources to the microbiota (Fig. 2). Denitrification was previously shown to occur in the Mediterranean sponge species *Dysidea avara* and *Chondrosia reniformis* [97]. Denitrification can be favored over other nitrogen cycling pathways in anaerobic niches within the sponge tissue, possibly resulting in nitrogen-limited conditions to the microbiota. Under these conditions, MeA may serve as a nitrogen source to part of the microbial community and the process would result in methane release. Future work involving functional characterization of sponge-hosted *aat* homologues is needed to test our proposed involvement of *aat* in sponge-associated aerobic methane synthesis.

### Methylphosphonate and methylamine sources in marine sponges

The source of MPN and MeA may be exogenous, with the sponge concentrating these compounds from DOM during its efficient water filtration. MPN and MeA have in fact previously been reported as common compounds in seawater [56, 98–100]. Alternatively, these substrates for methane generation may be derived from endogenous metabolism. Based on our inability to detect methylphosphonate synthase (*mpnS*) genes or transcripts in our data, encoding the enzyme responsible for MPN synthesis [101], we propose that MPN is not produced endogenously by the sponge-associated community and rather likely sourced from the surrounding seawater. Conversely, the source of MeA may indeed be endogenous, that is, derived from the metabolic processing of TMA [102]. TMA can be produced from choline [103], glycine betaine [104], or carnitine [105]. Genes and transcripts related to TMA generation from glycine (*grdH*)- and choline (*cutC*)*-*derived transformations were not detected in our genomes and metatranscriptomes. We, however, report the presence of gene homologues to *cntAB*, involved in carnitine-based TMA biosynthesis [105]. We performed a metabolomic analysis which directly detected the presence of carnitine in our *A. aerophoba* tissue samples (Supplemental File 1). Carnitine (γ-trimethylamino-β-hydroxybutyric acid) is a ubiquitous quaternary amine produced by all members of animalia, including Porifera [106] that is metabolized by prokaryotes under aerobic conditions into TMA and malic acid or, under anaerobic conditions, to glycine betaine and, subsequently, glycine [107]. Recent studies of *A. aerophoba* used metagenomics and single cell sequencing to infer the presence of a symbiont guild specialized in carnitine utilization as (i) a source of carbon and nitrogen anabolism [39] and/or (ii) a substrate for energy-yielding catabolism [18]. Furthermore, the presence of 2-methylbutyryl-carnitine was reported for six sponge species from the Great Barrier Reef, Australia [108], supporting the ubiquitous presence of carnitine-related compounds in sponges and their potential not only as a source of food but also as precursor to methane generating substrates for the associated microbiota. In fact, microbial-mediated production of TMA from carnitine in our samples is predicted by the transcription of the *cntAB* genes by lineages classified as Alphaproteobacteria and Actinobacteria (Fig. 1). Furthermore, TMA oxidation to trimethylamine-N-oxide (TMAO) and “back-production” from TMAO are predicted by the detection of FMO transcripts from Proteobacteria and Actinobacteria and *torA* genes in Actinobacteria, encoding TMA oxidase [109] and TMO reductase [110], respectively. The detection of genes homologous to *tdm* and at least one subunit of the *dmmABC* complex in Alphaproteobacterial lineages, encoding TMAO demethylase [109] and DMA monooxygenase [111], respectively, also suggests that TMAO may be further oxidized to dimethylamine (DMA) and, finally, MeA (Fig. 1). Taken together, we suggest that, while both substrates for aerobic bacterial methane synthesis (MPN and MeA) may be seawater derived, MeA may also be endogenously produced through the recycling of sponge cell debris (i.e., carnitine) resulting from continuous replacement of choanocyte cells. A high turnover of choanocyte cells, involving high proliferation followed by cell shedding, was shown to occur in *Halisarca caerulea* and was suggested to enable the constant renewal of the sponge filter system required for its efficient water filtration [112].

### Deltaproteobacteria (Binatota) methylotrophs as potential methane sinks

**Fig. 4:**
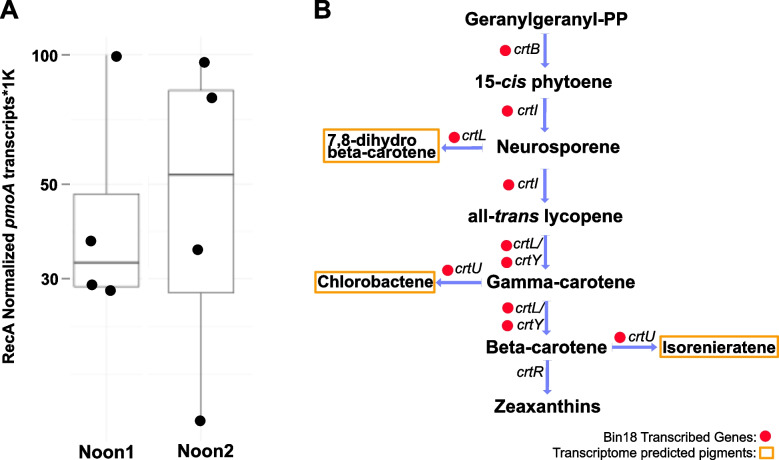
Transcription of Deltaproteobacterial (bin18, Candidate Phylum Binatota) *pmoA* gene and pigment biosynthesis pathways. **A** *recA* normalized transcriptional activity of *pmoA* gene in four sponge specimens and two time points. **B** Transcribed genes related to pigment biosynthesis pathways identified in the Deltaproteobacterial MAG are denoted by red circles next to the gene names. Predicted pigment metabolites from this activity are highlighted in yellow boxes

With two potential sources of biogenic aerobic methane in the sponge holobiont, the highly active *A. aerophoba*-associated symbiont bin18 (Candidate phylum Binatota, or Desulfobacterota, an unclassified Deltaproteobacteria lineage according to NCBI taxonomy) is here identified as a likely methane sink (Figs. 1 and S2). A previous detailed study of this lineage described them as pigment production specialists with a predicted lifestyle that is heavily reliant on aerobic methylotrophy and alkane degradation [113]. We show that bin18 actively transcribes methane monooxygenase (*pmoA*) gene (Fig. 4A). Similarly, a member of the same phylum was recently reported to express *pmoA* in the sponge species *P. ficiformis* [42] implicating this Deltaproteobacterial (Binatota) lineage in sponge methane oxidation. Methylotrophy results in reducing equivalents through the formation of methanol as a key intermediate [114] and can thus facilitate the production of carotenoid pigments. Furthermore, we detect the transcription of genes involved in all the intermediate steps necessary for 7, 8-dihydro beta-carotene, chlorobactene, and isorenieratene pigment biosynthesis (Fig. 4B). These pigments can serve as photoprotectants for the host and its symbionts [115]. Together, our results suggest that at least two independent methane production pathways, using host-derived MeA and seawater-derived MPN, represent previously unrecognized syntrophies that, through methane as an intermediate, contribute to the production of photoprotective pigments that may benefit the sponge holobiont.

### Global distribution of sponge-associated methylotrophs

**Fig. 5:**
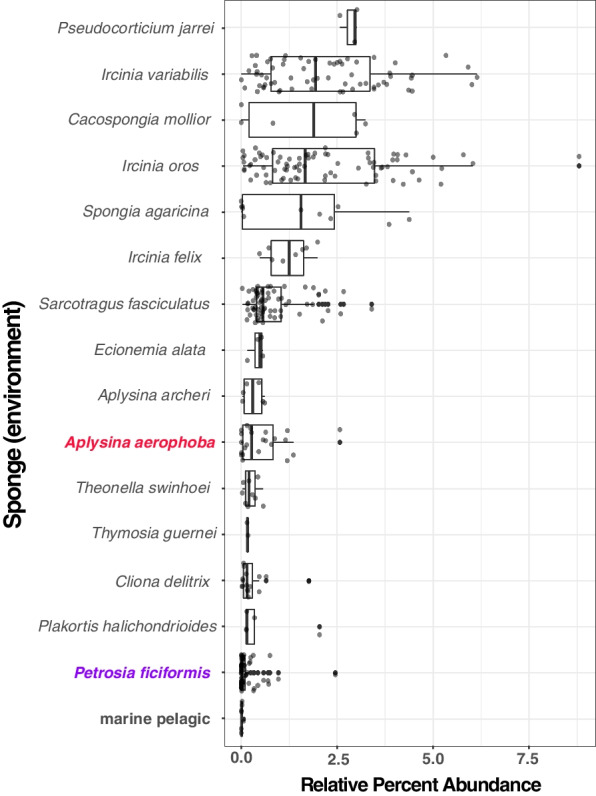
Global sponge-associated Binatota survey. Relative percent abundance of 16S rRNA sequences with >98% sequence ID and 99% of query length to the *A. aerophoba* bin18 (Binatota) 16S rRNA gene sequence, summarized in quartile boxplots. Species shown had significantly (Student *t*-test, *P*_val_ < 0.05) higher relative abundances of bin18 16S rRNA gene matches than marine pelagic metagenomes. Quartile box plots are arranged in descending median values of all observations from top to bottom. *Aplysina aerophoba* and *Petrosia ficiformis*, both sponges analyzed in this study, are highlighted in red and purple, respectively

To determine the global prevalence of sponge-associated methylotrophic pigment producers, we searched the Sponge Microbiome Project (SMP) dataset for highly similar (>98% ID, >99% subject length alignment) matches to the 16S rRNA gene sequence recovered from Binatota (bin18). We identified numerous matches to our Binatota query sequence in at least 46 sponge species (Fig. S7) with significant (Student *t*-test, *P*_val_ < 0.05) enrichment of this lineage in 15 sponge species relative to seawater metagenomes (Fig. 5). The highest percent Binatota abundances in the SMP dataset are observed in the following sponge species: *Pseudocorticium jarrei*,* Ircinia variabilis*,* Cacospongia mollior*,* Ircinia oros*, and *Spongia agaricina*. We note that Binatota phylotypes are significantly enriched in *A. aerophoba* and *Aplysina archeri* specimens relative to seawater (Fig. 5); however, this enrichment is not detected in six other *Aplysina* species sampled (Fig. S7). This suggests that microbial methane cycling potential may be a species-specific activity in the genus *Aplysina*. Interestingly, we found that *P. ficiformis*, another Mediterranean sponge species with recently published metatranscriptomes, metagenomes, and MAGs [42, 60], also hosts a significantly enriched Binatota community. For this reason, we used *P. ficiformis* as a second model for assessing methane cycling potential in sponges.

### Methane cycling meta-analysis of *Petrosia ficiformis*

To explore whether MPN- and MeA-based methane cycling activity is unique to *A. aerophoba*, or rather a more widely distributed characteristic of sponge holobionts, we re-analyzed recently published collections of MAGs and metatranscriptomes of the species *P. ficiformis* [42, 60]. Genes involved in carnitine breakdown to methylamines (*cntAB*, *tdm*, *dmmB*), MeA-based methane generation (*aat*) [59], and methane oxidation (*pmoA*) were detected in *P. ficiformis* MAGs (Fig. S8). In both sponge species, MeA production potential is predicted predominantly from Alphaproteobacterial community members. Additional predicted aerobic methane producing lineages in both sponge species include members of the Actinobacteria, while in *P. ficiformis*, Bacteroides, Latescibacteria, and Chloroflexi may also be involved in this activity. The gene marker for MPN-based methane production (*phnJ*), present and actively transcribed in *A. aerophoba* symbionts (Fig. 1), was not detected in *P. ficiformis* MAGs nor was it found expressed based on its metatranscriptomes. These results indicate that the bioavailability of phosphate to symbionts may differ across demosponge species. Lastly, we note that *pmoA* gene homologues and transcripts, indicative of methane oxidation, were also detected in a Deltaproteobacterial MAG, classified as Candidate Phylum Binatota, from *P. ficiformis* [42]. These meta-analysis results show that methane cycling may be widespread among Porifera. While the Binatota appear to serve as a common methane sink in both *A. aerophoba* and *P. ficiformis*, the microbial phyla responsible for methane generation and the pathways involved, appear to be species-specific (Figs. 1 and S8).

### Local and global ecological impact of methane cycling in marine sponges

**Fig. 6:**
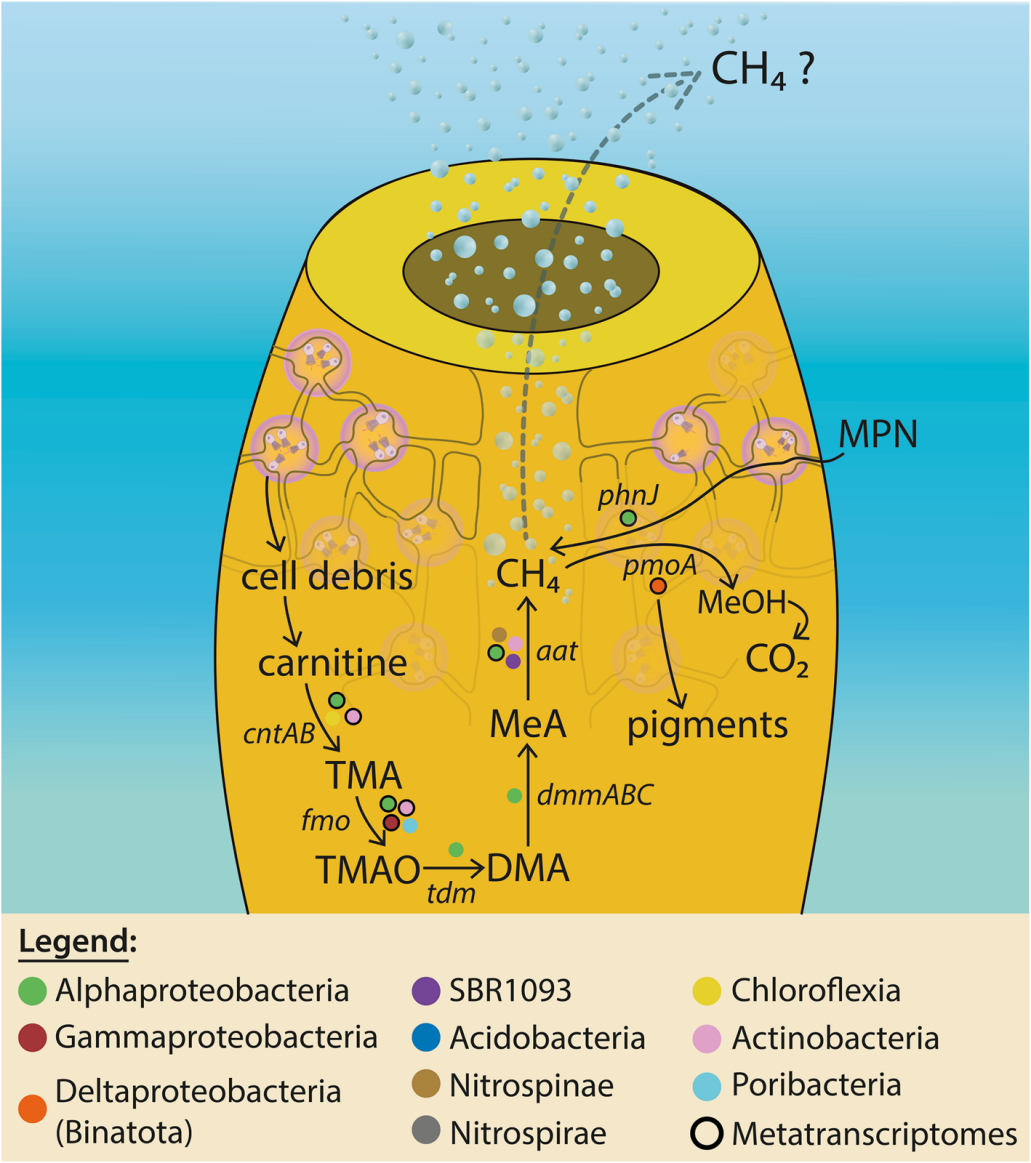
Bacterial aerobic methane cycling model. Conceptual diagram depicting a metabolic cascade involved in major microbial transformations leading to methane production/oxidation. Genes for key enzymes involved in each substrate transformation are denoted in italics. The phylogenetic association of bins where they have been detected are shown as color-coded circles next to reaction arrows. Circles with black frame also recruited transcript reads for the depicted marker gene, meaning the function was active at the time of sampling

Symbionts enable their animal hosts to benefit from microbial metabolism and, ultimately, impact ecosystem health and function [2]. We show one such case study involving the biogeochemical cycling of C, N, and P, via two independent pathways for aerobic bacterial methane synthesis, to produce photoprotectant pigments that directly benefit the sponge host: host-derived organic matter (carnitine) **+** environmentally filtered compounds (e.g., MPN) → in situ aerobic bacterial methane production + generation of bioavailable N and P for symbionts + photoprotective pigments for host (Fig. 6).

Methane is a potent greenhouse gas capable of trapping 3.7 times more radiated heat than CO_2_ [116] and a significant determinant, even in trace amounts, of the Earth’s radiative atmospheric balance [117]. The ocean is an important source of global methane emissions [118, 119]. Depending on the coupling of methanogenesis and methanotrophy in sponges, methane may be internally cycled, consumed, or emitted to the surrounding seawater. If emitted, considering the over half-billion-year-old history of sponge symbiosis [3, 4] and the remarkable water filtration activity of marine sponges [7], it is possible that this previously unrecognized marine animal-hosted methane cycle may have, over geological time, influenced methane concentrations of marine environments and possibly fluxes of methane from supersaturated ocean waters to the atmosphere.

## Conclusions

The metabolic activities of the sponge microbiome are saliently diverse; interestingly, here, we also predict aerobic bacterial methane synthesis based on independent methylphosphonate and methylamine metabolisms. Together with the presence of abundant and active methylotrophic community members, this suggests the existence of a previously unrecognized aerobic bacterial methane cycle in demosponges that may affect methane concentration in sponge-dominated marine habitats. Further studies including quantitative methane measurements in sponge incurrent and excurrent seawater, in the presence of natural and amended methanogenic substrates (MeA and MPN), and under different nutrient (N and P) conditions, will enhance our understanding of the influence of sponge holobionts on present and future marine ecosystems.

## Supplementary Information

**Additional file 1: Figure S1.** Amplicon Sequence Variants (ASVs) community composition and activity based on 16S rRNA genes amplified using as template either genomic DNA (gDNA, panel A) or complementary DNA (cDNA; synthesized from RNA; panel B) from the same four sponge specimens used in the metatranscriptomics libraries. Color codes represent the relative (A) community composition and (B) a proxy for the transcriptional activity of prokaryotic lineages classified at the Class-level of taxonomy. Each sponge specimen was sampled 24h apart at noon. **Figure S2.** Abundance normalized activity survey for 37 dominant lineages, based on metatranscriptomic read mapping against MAGs, shown for all four sponge replicates sampled at noon 24h apart. Data is arranged based on phylogenomic relatedness and color-coded at the Phylum-level of taxonomy, in the *A. aerophoba*-associated microbiome. Relative activity is represented by circle size and also color-coded based onthe Phylum-level association of each depicted lineage. **Figure S3. **Complete KEGG-decoder pathway estimates heatmaps for all metatranscriptomic samples. Additional functional categories shown here and not in Figure 1 include: O_2_ cytochromes, hydrogen cycle, vitamins and transporters, secretion systems, and amino acid synthesis. **Figure S4.** *mttB* gene transcript homologue alignment localized to a window between residues 303-440. The red column highlights residue 333, an expected non-conical pyrrolysine “O” residue found in all functional trimethylamine-corrinoid protein co-methyltransferases and absent in the sponge bacterial homologues. **Figure S5.** Delta-BLAST analysis for conserved functional motifs in proteins predicted from transcribed *aat *gene homologues. **Figure S6. **Annotated version of phylogenetic tree of *aat *genes and transcripts presented in Fig. 3. **Figure S7.** Relative percent abundance of 16S rRNA sequences with >98% sequence ID and 99% of query length to the *A. aerophoba *bin18 (Binatota) 16S rRNA gene sequence, summarized in quartile boxplots. Quartile box plots are arranged in descending median values of all observations from top to bottom. *Aplysina aerophoba *and *Petrosia ficiformis*, both sponges analyzed in this study, are highlighted in red and purple, respectively. Single and double asterisks depict sponge species with significantly (student t-test, P_val_ < 0.05) higher abundance of bin18-related sequences than in marine pelagic and marine sediment 16S rRNA datasets, respectively. **Figure S8.** Meta-analysis of methane-cycling genes using public MAGs (arranged based on phylogenomic relatedness, (Burgsdorf et al. 2022) and metatranscriptomes from Mediterranean *P. ficiformis *sponges (Britstein et al. 2020). (A) Detected methane-related genes (black circles) and associated transcripts (red circles) for a collection of sponge symbiont MAGs. (B) Alignment metrics of metatranscriptome predicted (Transcript_ID) and MAG retrieved (Protein_ID) protein sequences identified as *aat *homologues including query (Tlen) and sequence length (Plen), %ID, Eval, and presence or absence of conserved domains. (C) Delta-BLASTp summaries of conserved domain position along a representative transcribed *aat *homologue sequence (all transcribed *aat *homologues shown in panel A contained identical conserved domain positions). **TableS1.** Metatranscriptomic read retention percentages following adapter trimming, interleaving, and rRNA library alignments for *in silico *rRNA depletion, prior to *de novo *assembly using rnaSPAdes.

## Data Availability

All metatranscriptomes used in this study are publicly available under the following NCBI Bio project ID: PRJNA893080 and SRA accession numbers: SRX17994477-SRX17994508.

## Abbreviations

3HP: 3-Hydroxypropionate
4HB/3HP: 4-Hydroxybutyrate/3-hydroxypropionate
AAT: Aspartate aminotransferase
CBB: Calvin-Benson-Bassham
DMA: Dimethylamine
HMA: High microbial abundance
HMM: Hidden Markov model
Lys: Lysine
MAGs: Metagenome-assembled genomes
*mcrA*: Methyl-coenzyme M reductase
MeA: Methylamine
MPN: Methylphosphonate
*mpnS*: Methylphosphonate synthase
*mttB*: Methyltransferase
QC: Quality control
rTCA: Reductive tricarboxylic acid
SMP: Sponge microbiome project
SRBs: Sulfate-reducing bacteria
TMA: Trimethylamine
TMAO: Trimethylamine-N-oxide

## References

[1] Thomas T (2016). Diversity, structure and convergent evolution of the global sponge microbiome. Nat Commun.

[2] Pita L (2018). The sponge holobiont in a changing ocean: from microbes to ecosystems. Microbiome.

[3] Brain CKB, et al. The first animals: ca. 760-million-year-old sponge-like fossils from Namibia. S Afr J Sci. 2012;108(1-2):01–8.

[4] Li C, Chen Y, Hua T (1998). Precambrian sponges with cellular structures. Science.

[5] Feuda R (2017). Improved modeling of compositional heterogeneity supports sponges as sister to all other animals. Curr Biol.

[6] Hentschel U (2012). Genomic insights into the marine sponge microbiome. Nat Rev Microbiol.

[7] Morganti TM (2019). Size is the major determinant of pumping rates in marine sponges. Front Physiol.

[8] Yahel G (2003). In situ feeding and element removal in the symbiont-bearing sponge Theonella swinhoei: bulk DOC in the major source of carbon. Limnol Oceanogr.

[9] Rooks C (2020). Deep-sea sponge grounds as nutrient sinks: denitrification is common in boreo-Arctic sponges. Biogeosciences.

[10] Rix L (2016). Coral mucus fuels the sponge loop in warm- and cold-water coral reef ecosystems. Sci Rep.

[11] Goeij J (2013). Surviving in a marine desert: the sponge loop retains resources within coral reefs. Science.

[12] Kahn AS (2015). Benthic grazing and carbon sequestration by deep-water glass sponge reefs. Limnol Oceanogr.

[13] Zhang F (2015). Phosphorus sequestration in the form of polyphosphate by microbial symbionts in marine sponges. Proc Natl Acad Sci U S A.

[14] Gloeckner V (2014). the HMA-LMA dichotomy revisited: an electron microscopical survey of 56 sponge species. Biol. Bull..

[15] Wilkinson C (1978). Microbial associations in sponges. III. Ultrastructure of the in situ associations in coral reef sponges. Mar Biol.

[16] Hentschel U, Usher KM, Taylor MW (2006). Marine sponges as microbial fermenters. FEMS Microbiol Ecol.

[17] Lackner G (2017). Insights into the lifestyle of uncultured bacterial natural product factories associated with marine sponges. Proc Natl Acad Sci U S A.

[18] Bayer K (2018). Marine sponges as Chloroflexi hot spots: genomic insights and high-resolution visualization of an abundant and diverse symbiotic clade. mSystems.

[19] Horn H (2016). An enrichment of CRISPR and other defense-related features in marine sponge-associated microbial metagenomes. Front Microbiol.

[20] Burgsdorf I (2015). Lifestyle evolution in cyanobacterial symbionts of sponges. mBio.

[21] Thomas T (2010). Functional genomic signatures of sponge bacteria reveal unique and shared features of symbiosis. ISME J.

[22] Webster NS, Thomas T (2016). The sponge hologenome. mBio.

[23] Haber M (2020). Genomic insights into the lifestyles of Thaumarchaeota inside sponges. Front Microbiol.

[24] Bayer K, Schmitt S, Hentschel U (2008). Physiology, phylogeny and in situ evidence for bacterial and archaeal nitrifiers in the marine sponge Aplysina aerophoba. Environ Microbiol.

[25] Moeller FU (2019). Characterization of a thaumarchaeal symbiont that drives incomplete nitrification in the tropical sponge Ianthella basta. Environ Microbiol.

[26] Camargo JA, Alonso A (2006). Ecological and toxicological effects of inorganic nitrogen pollution in aquatic ecosystems: a global assessment. Environ Int.

[27] de Goeij JM (2008). Tracing 13C-enriched dissolved and particulate organic carbon in the bacteria-containing coral reef sponge Halisarca caerulea: evidence for DOM-feeding. Limnol Oceanogr.

[28] Jiménez E, Ribes M (2007). Sponges as a source of dissolved inorganic nitrogen: nitrification mediated by temperate sponges. Limnol Oceanogr.

[29] Hoffmann F (2009). Complex nitrogen cycling in the sponge Geodia barretti. Environ Microbiol.

[30] Yahel G (2007). In situ feeding and metabolism of glass sponges (Hexactinellida, Porifera) studied in a deep temperate fjord with a remotely operated submersible. Limnol Oceanogr.

[31] Ribes M (2012). Functional convergence of microbes associated with temperate marine sponges. Environ Microbiol.

[32] Maldonado M (2019). Sponge skeletons as an important sink of silicon in the global oceans. Nat Geosci.

[33] Morganti T, et al. VacuSIP, an improved InEx method for in situ measurement of particulate and dissolved compounds processed by active 919 suspension feeders. J Vis Exp. 2016;(114):54221. 10.3791/54221 PMC5091718 27585354

[34] Yahel G, Marie D, Genin A (2005). InEx-a direct in situ method to measure filtration rates, nutrition, and metabolism of active suspension feeders. Limnol Oceanogr Methods.

[35] Zhang F (2019). Microbially mediated nutrient cycles in marine sponges. FEMS Microbiol Ecol.

[36] Maldonado M, Ribes M, van Duyl FC (2012). Nutrient fluxes through sponges: biology, budgets, and ecological implications. Adv Mar Biol.

[37] Rix L (2020). Heterotrophy in the earliest gut: a single-cell view of heterotrophic carbon and nitrogen assimilation in sponge-microbe symbioses. ISME J.

[38] Hudspith M (2021). Subcellular view of host-microbiome nutrient exchange in sponges: insights into the ecological success of an early metazoan-microbe symbiosis. Microbiome.

[39] Slaby BM (2017). Metagenomic binning of a marine sponge microbiome reveals unity in defense but metabolic specialization. ISME J.

[40] Robbins SJ (2021). A genomic view of the microbiome of coral reef demosponges. ISME J.

[41] Moitinho-Silva L (2017). Integrated metabolism in sponge-microbe symbiosis revealed by genome-centered metatranscriptomics. ISME J.

[42] Burgsdorf I (2022). Lineage-specific energy and carbon metabolism of sponge symbionts and contributions to the host carbon pool. ISME J.

[43] Radax R (2012). Metatranscriptomics of the marine sponge Geodia barretti: tackling phylogeny and function of its microbial community. Environ Microbiol.

[44] Taylor JA (2021). Phylogeny resolved, metabolism revealed: functional radiation within a widespread and divergent clade of sponge symbionts. ISME J.

[45] Fan L (2013). Marine microbial symbiosis heats up: the phylogenetic and functional response of a sponge holobiont to thermal stress. ISME J.

[46] Webster NS (2001). Phylogenetic diversity of bacteria associated with the marine sponge Rhopaloeides odorabile. Appl Environ Microbiol.

[47] Hoffmann F (2008). Oxygen dynamics and transport in the Mediterranean sponge Aplysina aerophoba. Mar Biol.

[48] Lavy A (2016). Intermittent hypoxia and prolonged suboxia measured in situ in a marine sponge. Front Mar Sci.

[49] Hoffmann F (2005). An anaerobic world in sponges. Geomicrobiol J.

[50] Said Hassane C (2020). Microorganisms associated with the marine sponge Scopalina hapalia: a reservoir of bioactive molecules to slow down the aging process. Microorganisms.

[51] Rubin-Blum M (2017). Short-chain alkanes fuel mussel and sponge Cycloclasticus symbionts from deep-sea gas and oil seeps. Nat Microbiol.

[52] Vacelet J, Boury-Esnault N (2002). A new species of carnivorous deep-sea sponge (Demospongiae: Cladorhizidae) associated with methanotrophic bacteria. Cah Biol Mar.

[53] Vacelet J (1995). A methanotrophic carnivorous sponge. Nature.

[54] Rubin-Blum M (2019). Fueled by methane: deep-sea sponges from asphalt seeps gain their nutrition from methane-oxidizing symbionts. ISME J.

[55] Murphy C (2021). Genomic analysis of the yet-uncultured Binatota reveals broad methylotrophic, alkane-degradtion, and pigment production capacities. mBio.

[56] Repeta DJ (2016). Marine methane paradox explained by bacterial degradation of dissolved organic matter. Nat Geosci.

[57] Metcalf WW (2012). Synthesis of methylphosphonic acid by marine microbes: a source for methane in the aerobic ocean. Science.

[58] Langmead B, Salzberg SL (2012). Fast gapped-read alignment with Bowtie 2. Nat Methods.

[59] Wang Q (2021). Aerobic bacterial methane synthesis. Proc Natl Acad Sci U S A.

[60] Britstein M (2020). Sponge microbiome stability during environmental acquisition of highly specific photosymbionts. Environ Microbiol.

[61] Kopylova E, Noe L, Touzet H (2012). SortMeRNA: fast and accurate filtering of ribosomal RNAs in metatranscriptomic data. Bioinformatics.

[62] Bushmanova E (2019). rnaSPAdes: a de novo transcriptome assembler and its application to RNA-Seq data. Gigascience.

[63] Callahan BJ (2016). DADA2: High-resolution sample inference from Illumina amplicon data. Nat Methods.

[64] Quast C (2013). The SILVA ribosomal RNA gene database project: improved data processing and web-based tools. Nucleic Acids Res.

[65] McMurdie PJ, Holmes S (2013). phyloseq: an R package for reproducible interactive analysis and graphics of microbiome census data. PLoS One.

[66] Hyatt D (2010). Prodigal: prokaryotic gene recognition and translation initiation site identification. BMC Bioinformatics.

[67] Kanehisa M (2016). KEGG as a reference resource for gene and protein annotation. Nucleic Acids Res.

[68] Kanehisa M, Sato Y, Morishima K (2016). BlastKOALA and GhostKOALA: KEGG tools for functional characterization of genome and metagenome sequences. J Mol Biol.

[69] Graham ED, Heidelberg JF, Tully BJ (2018). Potential for primary productivity in a globally-distributed bacterial phototroph. ISME J.

[70] Abrams ZB (2019). A protocol to evaluate RNA sequencing normalization methods. BMC Bioinformatics.

[71] Lee MD (2019). GToTree: a user-friendly workflow for phylogenomics. Bioinformatics.

[72] Edgar RC (2004). MUSCLE: multiple sequence alignment with high accuracy and high throughput. Nucleic Acids Res.

[73] Capella-Gutierrez S, Silla-Martinez JM, Gabaldon T (2009). trimAl: a tool for automated alignment trimming in large-scale phylogenetic analyses. Bioinformatics.

[74] Price MN, Dehal PS, Arkin AP (2010). FastTree 2–approximately maximum-likelihood trees for large alignments. PLoS One.

[75] Seemann T (2014). Prokka: rapid prokaryotic genome annotation. Bioinformatics.

[76] Kind T, Fiehn O (2010). Advances in structure elucidation of small molecules using mass spectrometry. Bioanal Rev.

[77] Kind T (2009). FiehnLib: mass spectral and retention index libraries for metabolomics based on quadrupole and time-of-flight gas chromatography/mass spectrometry. Anal Chem.

[78] Feng G (2019). Analysis of functional gene transcripts suggests active CO2 assimilation and CO oxidation by diverse bacteria in marine sponges. FEMS Microbiol Ecol.

[79] Kamke J (2013). Single-cell genomics reveals complex carbohydrate degradation patterns in poribacterial symbionts of marine sponges. ISME J.

[80] Tanaka T (2004). Concerted action of diacetylchitobiose deacetylase and exo-beta-D-glucosaminidase in a novel chitinolytic pathway in the hyperthermophilic archaeon Thermococcus kodakaraensis KOD1. J Biol Chem.

[81] Fiore CL (2020). Trait-based comparison of coral and sponge microbiomes. Sci Rep.

[82] Tian RM (2017). Genome reduction and microbe-host interactions drive adaptation of a sulfur-oxidizing bacterium associated with a cold seep sponge. mSystems.

[83] Tian RM (2014). Genomic analysis reveals versatile heterotrophic capacity of a potentially symbiotic sulfur-oxidizing bacterium in sponge. Environ Microbiol.

[84] Jensen S (2017). The relative abundance and transcriptional activity of marine sponge-associated microorganisms emphasizing groups involved in sulfur cycle. Microb Ecol.

[85] Canfield DE, Teske A (1996). Late proterozoic rise in atmospheric oxygen concetration inferred from phylogenetic and sulphur-isotope studies. Nature.

[86] Moran NA, McCutcheon JP, Nakabachi A (2008). Genomics and evolution of heritable bacterial symbionts. Annu Rev Genet.

[87] Fiore CL (2015). Transcriptional activity of the giant barrel sponge, Xestospongia muta Holobiont: molecular evidence for metabolic interchange. Front Microbiol.

[88] Usher K (2008). The ecology and phylogeny of cyanobacterial symbionts in sponges. Mar Ecol.

[89] Freeman CJ, Thacker RW (2011). Complex interactions between marine sponges and their symbiotic microbial communities. Limnol Oceanogr.

[90] Brugere JF (2014). Archaebiotics: proposed therapeutic use of archaea to prevent trimethylaminuria and cardiovascular disease. Gut Microbes.

[91] Rother M, Krzycki JA (2010). Selenocysteine, pyrrolysine, and the unique energy metabolism of methanogenic archaea. Archaea.

[92] Ticak T (2014). A nonpyrrolysine member of the widely distributed trimethylamine methyltransferase family is a glycine betaine methyltransferase. Proc Natl Acad Sci U S A.

[93] Karl DM (2008). Aerobic production of methane in the sea. Nat Geosci.

[94] Sosa OA (2019). Phosphate-limited ocean regions select for bacterial populations enriched in the carbon-phosphorus lyase pathway for phosphonate degradation. Environ Microbiol.

[95] Fiore CL, Baker DM, Lesser MP (2013). Nitrogen biogeochemistry in the Caribbean sponge, Xestospongia muta: a source or sink of dissolved inorganic nitrogen?. PLoS One.

[96] Mohamed NM (2008). Diversity and expression of nitrogen fixation genes in bacterial symbionts of marine sponges. Environ Microbiol.

[97] Schlappy ML (2010). Evidence of nitrification and denitrification in high and low microbial abundance sponges. Mar Biol.

[98] Clark L, Ingall E, Benner R (1999). Marine organic phosphorous cycling: novel insights from nuclear magnetic resonance. Am J Sci.

[99] Cree CHL (2018). Measurement of methylamines in seawater using solid phase microextraction and gas chromatography. Limnol Oceanogr Methods.

[100] Mausz MA, Chen Y (2019). Microbiology and ecology of methylated amine metabolism in marine ecosystems. Curr Issues Mol Biol.

[101] Born D (2017). Structural basis for methylphosphonate biosynthesis. Science.

[102] Loo RL (2022). Balancing the equation: a natural history of trimethylamine and trimethylamine-N-oxide. J Proteome Res.

[103] Martinez-del Campo A (2015). Characterization and detection of a widely distributed gene cluster that predicts anaerobic choline utilization by human gut bacteria. mBio.

[104] Li L (2021). Bacteria and Archaea synergistically convert glycine betaine to biogenic methane in the Formosa cold seep of the South China Sea. mSystems.

[105] Zhu Y (2014). Carnitine metabolism to trimethylamine by an unusual Rieske-type oxygenase from human microbiota. Proc Natl Acad Sci U S A.

[106] Fraenkel G (1954). The distribution of vitamin Bt (Carnitine) throughout the animal kingdom. Arch Biochem Biophys.

[107] Meadows JA, Wargo MJ (2015). Carnitine in bacterial physiology and metabolism. Microbiology (Reading).

[108] Zhang S (2022). Comparative metabolomic analysis reveals shared and unique chemical interactions in sponge holobionts. Microbiome.

[109] Chen Y (2011). Bacterial flavin-containing monooxygenase is trimethylamine monooxygenase. Proc Natl Acad Sci U S A.

[110] Méjean V (1994). TMAO anaerobic respiration in Escherichia coli: involvement of the tor operon. Molecular Microbiology.

[111] Lidbury I, Murrell JC, Chen Y (2014). Trimethylamine N-oxide metabolism by abundant marine heterotrophic bacteria. Proc Natl Acad Sci U S A.

[112] De Goeij JM (2009). Cell kinetics of the marine sponge Halisarca caerulea reveal rapid cell turnover and shedding. J Exp Biol.

[113] Murphy CL, Sheremet A, Dunfield PF, Spear JR, Stepanauskas R, Woyke T, et a. Genomic analysis of the yet-uncultured Binatota reveals broad methylotrophic, alkane-degradation, and pigment production capacities. mBio. 2021;12(3):e00985–21. 10.1128/mBio.00985-21 PMC8262859 34006650

[114] Chistoserdova L (2011). Modularity of methylotrophy, revisited. Environ Microbiol.

[115] Galasso C, Corinaldesi C, Sansone C (2017). Carotenoids from marine organisms: biological functions and industrial applications. Antioxidants (Basel).

[116] Lashof D, Dilip R (1990). Relative contributions of greenhouse gas emissions to global warming. Nature.

[117] Collins WJ (2018). Increased importance of methane reduction for a 1.5 degree target. Environ Res Lett.

[118] Rosentreter JA (2021). Half of global methane emissions come from highly variable aquatic ecosystem sources. Nat Geosci.

[119] Holmes ME (2000). Methane production, consumption, and air-sea exchange in the open ocean: an evaluation based on carbon isotopic ratios. Glob Biogeochem Cycles.

